# Multiomics Profiling Identifies *Tlr4* as a Therapeutic Target of Necroptosis in Spinal Cord Injury

**DOI:** 10.1155/mi/7306884

**Published:** 2026-07-24

**Authors:** Wanzhou Wang, Lu Sun, Wei Xie, Fangqing Chen, Cheng Hong

**Affiliations:** ^1^ Geriatric Hospital of Nanjing Medical University, Jiangsu Province Official Hospital, Nanjing, China, njmu.edu.cn; ^2^ Department of Pediatric Neurosurgery, Xinhua Hospital Affiliated to Shanghai Jiao Tong University School of Medicine, Shanghai, China, xinhuamed.com.cn

**Keywords:** bioinformatic analysis, necroptosis, spinal cord injury, *Tlr4*

## Abstract

Spinal cord injury (SCI) leads to a complex cascade of cellular events, among which necroptosis plays a critical role in exacerbating neuronal injury and inflammation. In this study, we aimed to identify and validate key genes associated with necroptosis in SCI using bulk RNA‐seq data, followed by differential analysis and weighted gene coexpression network analysis (WGCNA). We identified several candidate necroptosis‐related genes, and further least absolute shrinkage and selection operator (LASSO) regression highlighted five SCI‐necroptosis differentially expressed genes (DEGs): toll‐like receptor 4 (*Tlr4*), *Nlrp3*, *Il1b*, *Tnfaip3*, and *Stat4*. These genes were validated using RT‐qPCR and western blot experiments. Our analysis revealed that necroptosis scores were significantly elevated following SCI. Single‐cell RNA sequencing (scRNA‐seq) and spatial transcriptomics (ST) analysis revealed that *Tlr4* was upregulated in myeloid cells (microglia and macrophages) and played a pivotal role in triggering downstream necroptosis, which was confirmed by protein levels. In vitro and in vivo experiments confirmed that *Tlr4* inhibition attenuated necroptosis and inflammation. This study is the first to establish *Tlr4* as a direct upstream regulator of the pRIPK1/pRIPK3/pMLKL necroptotic axis in SCI, distinct from its role as a general inflammatory mediator, suggesting *Tlr4* as a promising therapeutic target for functional recovery.

## 1. Introduction

Spinal cord injury (SCI) is a devastating neurological condition that leads to permanent motor, sensory, and autonomic dysfunction, profoundly affecting patients’ quality of life [1, 2]. Despite advances in medical and surgical interventions, effective therapeutic strategies to promote functional recovery remain limited, largely due to the complex secondary injury mechanisms that exacerbate tissue damage and hinder repair [3]. Among these mechanisms, neuroinflammation plays a pivotal role in shaping the post‐injury microenvironment, with microglia, the resident immune cells of the central nervous system (CNS), emerging as key regulators of this process [4, 5]. Microglia are rapidly activated following SCI, whereas circulating monocytes are recruited to the lesion and differentiate into macrophages, together orchestrating the initiation and amplification of inflammatory responses [6]. Notably, these myeloid populations exhibit context‐dependent and often dual roles during injury progression. On the one hand, they contribute to tissue repair by clearing cellular debris, limiting secondary damage, and releasing trophic factors; on the other hand, sustained activation can drive excessive cytokine production, oxidative stress, and neuronal cell death, ultimately impairing functional recovery [7].

Necrosis and apoptosis are the main modes of cell death in SCI [8, 9]. Traditionally, necrosis was thought to be passive death unregulated by genes, but recent studies have shown that necrosis is also heavily regulated by genes in a form known as necroptosis [10]. Recent studies have highlighted necroptosis as a critical mediator for the activation of myeloid cells and neuroinflammation after SCI [11]. As a form of regulated necrosis characterized by membrane rupture and release of damage‐associated molecular patterns (DAMPs), necroptosis has been implicated in amplifying inflammatory responses and exacerbating tissue damage in various CNS injuries [12, 13]. However, the specific mechanisms by which necroptosis in microglia and macrophages contributes to SCI pathology remain poorly understood.

Toll‐like receptor 4 (*Tlr4*), a pattern recognition receptor widely expressed in microglia, has been identified as a key player in neuroinflammation and cell death pathways [14]. *Tlr4* activation by DAMP triggers downstream signaling cascades that promote inflammatory cytokine production and cell death [15, 16]. Notably, *Tlr4* has been linked to microglial necroptosis in various disease models and has shown its potential role in inhibiting necroptosis [17]. Although *Tlr4* has been extensively studied as an upstream regulator of inflammatory signaling in SCI, its potential role in necroptosis within myeloid cells has not been clearly defined.

In the present study, we utilized bioinformatics approaches to identify differentially expressed genes (DEGs) associated with necroptosis in SCI. We found that *Tlr4* plays a central role in regulating necroptosis in myeloid cells (microglia and macrophages). While previous studies have linked *Tlr4* to inflammation and cell death in CNS injury, they have largely treated *Tlr4* as a general inflammatory mediator. Here, for the first time, we establish *Tlr4* in myeloid cells as a direct upstream regulator of the pRIPK1‐pRIPK3‐pMLKL necroptotic axis in SCI, rather than merely a nonspecific inflammatory trigger. Through the inhibition of *Tlr4* in vitro and in vivo, we demonstrated that targeting this pathway could attenuate necroptosis, reduce inflammation, and promote neuron survival. Thus, our findings suggest that *Tlr4* is a promising target for therapeutic intervention in SCI, and further exploration of *Tlr4*‐mediated pathways may offer new insights into the development of effective strategies for SCI treatment and repair.

## 2. Materials and Methods

### 2.1. Data Collection and Processing

Open‐access microarray datasets were retrieved from the Gene Expression Omnibus (GEO) database at the National Center for Biotechnology Information (NCBI). Three bulk‐seq datasets, namely, GSE5296 (*n* = 96), GSE47681 (*n* = 34), and GSE42828 (*n* = 34), were selected for subsequent analysis. To address potential batch effects, the “ComBat” function from the sva package in R was employed. Following batch effect correction, principal component (PC) analysis (PCA) was performed on both the raw and batch‐corrected data to assess the efficacy of the batch effect removal process. Additionally, necroptosis‐related genes were systematically extracted from the Kyoto Encyclopedia of Genes and Genomes (KEGG) pathway database (https://www.kegg.jp/entry/mmu04217) to facilitate further investigation.

### 2.2. Weighted Gene Coexpression Network Analysis (WGCNA)

WGCNA is a pivotal tool in bioinformatics, widely recognized for its application in trait‐gene association studies. In the present study, the R package “WGCNA” was utilized to construct a coexpression network, with the consolidated gene expression data serving as the input and SCI/ sham as the trait data. Initially, sample clustering was performed using the hclust function to eliminate outlier samples, with the distance metric parameter set to “method = average.” Subsequently, an optimal soft threshold was determined to ensure the construction of a scale‐free network. Following this, a dynamic tree‐cutting algorithm was applied to identify the distinct modules. Module robustness was assessed using the “modulePreservation” function in WGCNA. The dataset was split into a 70% reference set and a 30% test set, and 200 permutations were performed. Modules with a *Z*‐summary > 10 were considered strongly preserved. Finally, Pearson’s correlation analysis was conducted to pinpoint modules significantly associated with SCI.

### 2.3. Selection of SCI‐ and Necroptosis‐Related DEGs (SCI‐Necroptosis DEGs)

The integrated expression matrix was subjected to differential expression analysis using the R package “limma,” with significance thresholds set at *p*  < 0.05 and |log2(FoldChange)| > 1. DEGs were visualized and identified through volcano plots. To pinpoint SCI‐related necroptosis genes, a three‐way intersection was performed among necroptosis‐related genes, SCI‐associated genes derived from WGCNA, and the identified DEGs. Subsequently, Gene Ontology (GO) and KEGG pathway enrichment analyses were conducted to elucidate the potential biological functions and pathways associated with the identified gene sets.

### 2.4. Phenotype Scoring of Necroptosis

To identify the SCI‐Necroptosis DEGs, least absolute shrinkage and selection operator (LASSO) regression were performed using the R package “glmnet.” Candidate genes from differential expression and WGCNA were input, and 10‐fold cross‐validation determined the optimal *λ*. The response type was configured as binomial, with the alpha parameter set to one. Model performance was assessed by calculating the area under the ROC curve (AUC) using the pROC package. To validate model stability, data were randomly split into a training set (70%) and validation set (30%), and the AUC was computed for both cohorts. Subsequently, necroptosis phenotypic scores were computed utilizing the single‐sample gene set enrichment analysis (ssGSEA) algorithm. Statistical analyses were then conducted to evaluate the differences in phenotypic scores between the sham and SCI groups as well as across distinct time points within the SCI group.

### 2.5. Construction of Interaction Networks

The protein–protein interaction (PPI) network among SCI‐necroptosis DEGs was constructed and visualized using the STRING database (https://string-db.org). Key genes identified through LASSO regression were designated as the central nodes of the PPI network, with the interaction confidence score threshold set to 0.4.

### 2.6. Immune Infiltration Analysis

Utilizing gene sets comprising 28 immune‐related cell types, the immune activity of each sample was assessed through the ssGSEA algorithm. The differences in immune infiltration between the two groups were quantified, and their correlations were systematically analyzed. Additionally, the relationship between immune infiltration levels and necroptosis expression was explored. Based on the median ssGSEA score, the SCI group was stratified into high‐ and low‐risk subgroups. GSEA was subsequently conducted to identify differential enrichment patterns between these subgroups. Significantly enriched gene sets were determined using stringent criteria, including |log2(FoldChange)| > 1 and a *p*‐value < 0.05.

### 2.7. Single‐Cell RNA Sequencing (scRNA‐seq) and Spatial Transcriptomics (ST) Analysis

GSE234774 (including scRNA‐seq and ST data) was processed and analyzed using the R package Seurat. For scRNA‐seq data, quality control measures were implemented with the following stringent criteria applied uniformly across all samples: (1) gene expression levels ranging between 200 and 10,000, (2) hemoglobin gene expression constituting less than 0.1% of total gene expression, and (3) mitochondrial gene expression accounting for less than 10% of total gene expression. Following quality filtering, the gene expression matrix was normalized and scaled, and the top 2000 highly variable genes were identified for subsequent analyses. Dimensionality reduction was conducted using PCA, with the first 30 PCs selected for clustering based on significant PCs determined through JackStraw analysis. Clustering was performed using the FindClusters function with a resolution parameter set to 0.8, and the resulting clusters were visualized in two‐dimensional space employing the uniform manifold approximation and projection (UMAP) method. Shared nearest neighbor (SNN) clustering was executed with a resolution of 0.8, followed by manual refinement of clusters based on canonical gene marker annotations. The “DimPlot” function was utilized to generate cluster visualization plots, while the “FeaturePlot” function was employed to visualize gene expression patterns across cells. To evaluate the spatially heterogeneous distribution of hub genes across tissue sections, we projected their expression onto the two‐dimensional spatial coordinates, thereby visualizing enrichment and gradient changes between the lesion core and surrounding regions.

### 2.8. Animals and SCI Model

Adult female C57BL/6J mice (8–10 weeks old, body weight 18–22 g) were purchased from the animal center of Nanjing Medical University (Nanjing, China). Mice were housed under specific pathogen‐free conditions at 22 ± 2°C with a 12 h light/dark cycle and had ad libitum access to food and water. Mice were randomly assigned to experimental groups using a random number generator. The sham group received a laminectomy only. For the SCI groups, mice were subjected to spinal cord contusion injury.

For SCI surgery, mice were anesthetized via an intraperitoneal injection of sodium pentobarbital (50 mg/kg). A surgical laminectomy was performed at the 10th thoracic vertebral level, and the T9–T10 spinal cord segment was carefully exposed. A standardized contusion injury was then induced using a spinal cord impactor device, in which a 5 g rod was dropped from a height of 10 cm onto the exposed spinal cord. The sham group underwent laminectomy only without spinal cord impact. Postoperative care included manual bladder expression twice daily until the recovery of normal micturition function. Mice with severe motor dysfunction not attributable to the injury (autotomy or infection) or unexpected death before the endpoint were excluded from the analysis.

### 2.9. Locomotor Function Recovery Assessment

The neurological function of the mice in each group was evaluated at specified time points following the procedure. The Basso Mouse Scale (BMS) score was used to assess all mice, focusing on hindlimb function, including coordination, ankle joint movement, weight support, plantar stepping, and trunk stability. The BMS score ranged from 0 (complete paralysis) to 9 (normal function).

To further evaluate the gross motor ability and coordination of the mice following SCI, each mouse underwent two assessments on an accelerating rotarod (0–40 rpm), with a 20 min interval between trials. The speed and duration of each trial were recorded and averaged for each mouse.

To assess hindlimb tactile sensitivity after SCI, the von Frey filament test was performed. Briefly, mice were placed on a metal grid, and calibrated von Frey filaments were applied to the plantar surface of the hind paw. Each paw received five consecutive stimuli. A positive response was defined as paw withdrawal or licking. The withdrawal threshold was defined as the minimal filament force eliciting at least three positive responses out of five trials.

### 2.10. RT‐qPCR

Total RNA was isolated from spinal cord tissues or myeloid cells using the TRIzol reagent according to the manufacturer’s protocol. The RNA was reverse‐transcribed into cDNA using a PrimeScript RT Reagent Kit, and RT‐qPCR was subsequently performed. Relative expression levels of target genes were normalized to reference genes and quantified using the 2^−ΔΔCT^ method. The primer sequences are listed in Supporting Information 1: Table S1.

### 2.11. Western Blot Analysis

Total protein was extracted from homogenized spinal cord tissues (collected from a 5 mm segment centered at the lesion epicenter, T8–T10) and from cultured myeloid cells. Proteins were denatured by boiling and separated using sodium dodecyl polyacrylamide gel electrophoresis, followed by membrane transfer and blocking. Primary antibodies were incubated overnight at 4°C. Secondary antibodies were incubated for 2 h at 25–30°C. Membranes were exposed using a Tanon 4600 chemiluminescence imaging system (Tanon Science, China). The antibodies used for immunoblotting in this study were anti‐β‐actin (Servicebio, 1:1000), anti‐pRIPK1 (Cell Signaling Technology, 1:1000), anti‐pRIPK3 (Cell Signaling Technology, 1:1000), anti‐pMLKL (Cell Signaling Technology, 1:1000), anti‐TLR4 (Servicebio, 1:500), anti‐NLRP3 (Servicebio, 1:500), anti‐IL‐1 beta (Abcam, 1:1000), anti‐TNFAIP3 (Servicebio, 1:1000), anti‐STAT4 (Abcam, 1:1000), anti‐MyD88 (Proteintech, 1:1000), anti‐p‐IκBα (Proteintech, 1:1000), and anti‐p‐p65 (Abcam, 1:1000).

### 2.12. Immunofluorescence

At the indicated time point, mice were deeply anesthetized with sodium pentobarbital and transcardially perfused with 50 mL of ice‐cold phosphate‐buffered saline (PBS), followed by 50 mL of 4% paraformaldehyde (PFA) in PBS. The spinal cord was carefully removed, and a 10 mm segment centered at the lesion epicenter (T8–T10) was collected. Tissues were postfixed in 4% PFA overnight at 4°C and then cryoprotected in 15% and 30% sucrose solutions at 4°C until they sank. The tissues were then embedded in an optimal cutting temperature (OCT) compound, frozen, and sectioned into 8 µm thick sections using a cryostat. Sections were washed with PBS, blocked with 5% normal serum for 1 h at room temperature, and incubated overnight at 4°C with primary antibodies: anti‐NeuN (Abcam, 1:1000) and anti‐CD68 (Abcam, 1:1000). After washing, sections were incubated with Alexa Fluor‐conjugated secondary antibodies (1:500) for 2 h at room temperature, followed by DAPI staining.

### 2.13. Isolation of Myeloid Cells From Mouse Spinal Cords

Spinal cord tissues were dissected from euthanized mice and subjected to mechanical and enzymatic dissociation using the Adult Brain Dissociation Kit (130‐107‐677, Miltenyi Biotec) following the manufacturer’s protocol. Tissue samples were enzymatically digested with collagenase IV (1 mg/mL) for 30 min at 37°C with gentle agitation. After digestion, the tissue was triturated to generate a single‐cell suspension and filtered through a 70 and 40 µm mesh to remove debris. Myelin removal was performed using Myelin Removal Beads II and Cell Debris Removal Solution according to the manufacturer’s instructions. For myeloid cell isolation, the cell suspension was incubated with anti‐CD11b microbeads (Miltenyi Biotec) for 30 min at 4°C with gentle rotation. The labeled cell suspension was then loaded onto a precooled MACS LS column placed in a magnetic field separator. CD11b+ myeloid cells were retained within the column, while unlabeled cells passed through. After washing, the column was removed from the magnetic field, and the magnetically retained myeloid cells were eluted by forceful flushing with ice‐cold PBS. Cell viability and concentration were determined using trypan blue exclusion and a hemocytometer.

### 2.14. Treatment of Primary Myeloid Cells

Primary myeloid cells were isolated and cultured in Dulbecco’s Modified Eagle Medium (DMEM) supplemented with 10% fetal bovine serum (FBS) and 1% penicillin streptomycin (P/S) solution at 37°C under 5% CO_2_ for maintenance. To induce necroptosis, myeloid cells were treated with TSZ according to the instructions of the Necroptosis Inducer Kit with TSZ (Beyotime). For rescue experiments, myeloid cells were treated with TSZ, followed by the addition of necrostatin‐1 (Nec‐1) (20 μM, a RIPK1 inhibitor, HY‐15760, MedChemExpress) or GSK‐872 (5 μM, a RIPK3 inhibitor, HY‐101872, MedChemExpress). To explore the role of *Tlr4*, myeloid cells were transfected with *Tlr4* siRNA or control siRNA (si Control) before TSZ treatment. The siRNA sequences and negative si Control were obtained from Sangon Biotech (Supporting Information 2: Table S2). Treated myeloid cells were then cocultured with primary neurons to investigate the potential interactions between activated myeloid cells and neurons.

### 2.15. Calcein‐Acetoxymethyl Ester (Calcein‐AM)/Propidium Iodide (PI) Double Staining

Cell viability and death were assessed using a dual staining approach with fluorescent probes. PI (Beyotime) was employed to identify dead cells through nuclear staining, while viable cells were quantified using Calcein‐AM (Beyotime), which is metabolized by living cells to produce green fluorescence. Following a 30 min incubation at 37°C in the dark, cells were washed twice with PBS to remove excess dye. Fluorescence images were captured using an inverted fluorescence microscope equipped with appropriate filter sets for PI (excitation/emission: 535/617 nm) and Calcein‐AM (excitation/emission: 494/517 nm). Quantitative analysis was performed by counting the number of Calcein‐AM‐positive (viable) and PI‐positive (dead) cells.

### 2.16. Statistical Analysis

Statistical analyses were performed using GraphPad Prism version 9.0. Differences between two groups were evaluated using a two‐tailed Student’s *t* test. Pearson correlation analysis was performed to examine the relationship between two continuous variables. For comparisons involving more than two factors, one‐way or two‐way analysis of variance (ANOVA) was carried out, with Bonferroni post hoc testing for multiple comparisons. Data were presented as mean ± standard deviation (SD), with statistical significance defined as *p* < 0.05.

## 3. Results

### 3.1. Establishment of WGCNA

**Figure 1:**
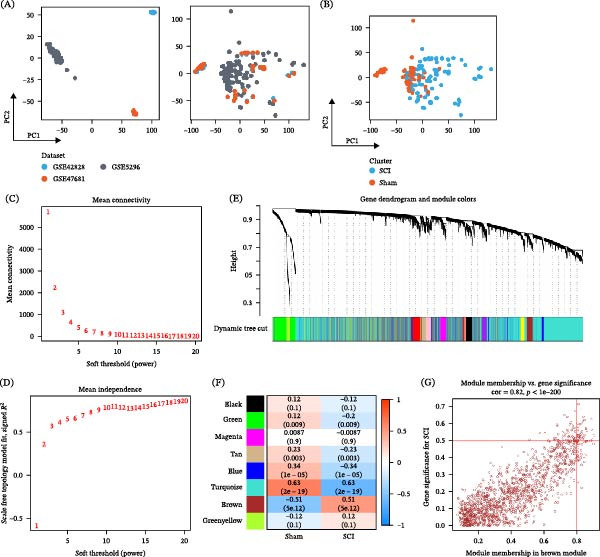
WGCNA reveals significant gene modules in SCI and sham groups across multiple datasets. (A) Principal component analysis (PCA) of three datasets (GSE5296, GSE47681, and GSE42828) before and after batch removal. (B) PCA analysis showing clustering of SCI and sham groups after batch removal. (C, D) Soft thresholding filtering for determining the optimal soft thresholding power (C) and scale free topology fit index (D) to construct a scale free network. (E) Dendrogram of gene clustering using hierarchical clustering with dynamic tree cut algorithm for module detection. Gene modules are color coded at the bottom. (F) Module–trait relationships table displaying the correlation between module membership and gene significance for each module. Color gradient represents the correlation coefficient. (G) Scatter plot of module membership versus gene significance for the brown module, demonstrating the relationship between gene significance and module membership for SCI.

The microarray datasets GSE42828, GSE47681, and GSE5296 were integrated based on their expression profiles (Figure 1A), with PCA demonstrating a clear separation between sham and SCI groups (Figure 1B). Differential gene expression analysis revealed distinct clustering patterns, enabling the identification of significant network characteristics.

For WGCNA, a soft threshold power (*β*) of 5 was selected based on the scale‐free topology criterion and mean connectivity analysis, with a correlation coefficient threshold of 0.75 established for module construction (Figure 1C, D). Application of the dynamic tree cut algorithm identified eight distinct gene modules (Figure 1E, F). Notably, the brown module demonstrated the strongest positive correlation with SCI (*R* = 0.51, *p* < 0.001). Further analysis revealed a highly significant relationship between module membership and gene significance within the brown module (cor = 0.82, *p* < 0.001) (Figure 1G). These findings suggest that genes within the brown module represent promising candidates for further investigation in SCI pathogenesis and therapeutic development.

### 3.2. Identification of DEGs and SCI–Necroptosis DEGs

**Figure 2:**
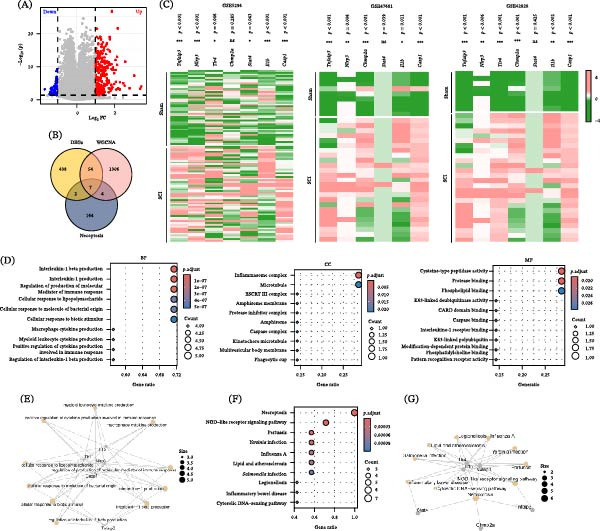
Differential gene expression and pathway analysis for SCI necroptosis related DEGs. (A) Volcano plot of DEG analysis, showing the significance (−log10(*p*‐value)) versus fold change (log2FC) for genes across different conditions. Red dots indicate significantly upregulated genes, blue dots indicate significantly downregulated genes, and gray dots indicate nonsignificant genes. (B) Venn diagram showing the overlap among DEGs, WGCNA identified genes, and necroptosis related genes. (C) Heatmap displaying gene expression patterns across three datasets (GSE5296, GSE47681, and GSE42828). (D) GO enrichment analysis for SCI necroptosis related DEGs in biological process (BP), cellular component (CC), and molecular function (MF) categories. (E) Network diagram showing the relationships between SCI necroptosis related DEGs and their associated GO terms, illustrating the interactions between genes and biological processes. (F) KEGG enrichment analysis for SCI necroptosis related DEGs. (G) Network diagram showing the relationships between SCI necroptosis related DEGs and KEGG pathways, with emphasis on inflammation and immune response related pathways. Data were analyzed with two‐tailed unpaired Student’s *t*‐test (C). Statistical significance is indicated as  ^∗^ *p* < 0.05,  ^∗∗^ *p* < 0.01, and  ^∗∗∗^ *p* < 0.001. ns indicates no significant difference (*p* > 0.05).

Differential expression analysis identified a total of 920 DEGs between SCI and sham groups, comprising 501 upregulated and 419 downregulated genes (Figure 2A). Through the intersection approach, we first identified overlapping genes between upregulated DEGs and the WGCNA brown module genes, followed by further intersection with necroptosis‐related genes. This analysis revealed seven SCI‐associated necroptosis DEGs (Figure 2B). The expression patterns of these genes were subsequently validated across three independent datasets using the Wilcoxon rank‐sum test, demonstrating consistent differential expression between SCI and sham groups (Figure 2C).

GO enrichment analysis of the SCI–necroptosis DEGs identified significant enrichment in inflammatory response pathways, including interleukin‐1 beta production, positive regulation of macrophage cytokine production, and cellular response to bacterial molecules (Figure 2D, E). These findings suggest a robust activation of immune response mechanisms following SCI. KEGG pathway analysis specifically focusing on SCI–necroptosis DEGs revealed significant involvement of the NOD‐like receptor signaling pathway, influenza A infection, and *Salmonella* infection (Figure 2F, G), providing mechanistic insights into necroptosis regulation in SCI pathogenesis.

### 3.3. Phenotype Scoring of Necroptosis

**Figure 3:**
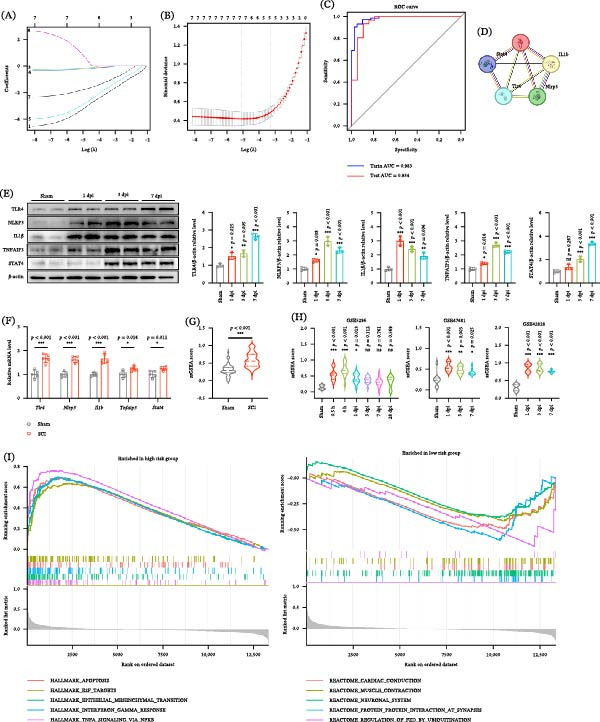
Selection and experimental verification of key gene. (A) LASSO regression plot showing the selection of key genes based on their coefficients across different lambda values. (B) Cross‐validation curve of LASSO regression model performance, showing the mean squared error (MSE) at each log(λ) value. (C) ROC curves of the LASSO model in the training and validation sets. (D) Gene network illustrating the interactions between selected genes based on protein–protein interactions (PPI). (E) Western blot for *Tlr4*, *Nlrp3*, *IL-1β*, *Tnfaip3*, and *Stat4* at different time points (*n* = 3 per group). (F) qRT‐PCR analysis of gene expression levels in sham and SCI groups, showing relative mRNA levels for *Tlr4*, *Nlrp3*, *Il-1β*, *Tnfaip3*, and *Stat4* (*n* = 5 per group). (G) Violin plot showing the distribution of single sample gene set enrichment analysis (ssGSEA) scores across SCI and sham groups. (H) Violin plot illustrating the distribution of ssGSEA scores across different time points in GSE5296, GSE47681, GSE42828. (I) GSEA of high and low risk groups based on key genes, showing the enrichment of inflammatory and immune response pathways. Data were analyzed with two‐tailed unpaired Student’s *t*‐test (F, G) and one‐way analysis of variance followed by post hoc Bonferroni correction (E, H). Statistical significance is indicated as  ^∗^ *p* < 0.05,  ^∗∗^ *p* < 0.01, and  ^∗∗∗^ *p* < 0.001.

LASSO regression analysis was employed to identify key predictive genes associated with SCI occurrence (Figure 3A–C). This analysis revealed five hub genes from the seven SCI‐necroptosis DEGs: *Tlr4*, *Nlrp3*, *Il1b*, *Tnfaip3*, and *Stat4*. A PPI network was constructed to elucidate the functional relationships among these hub genes, demonstrating significant molecular interactions (Figure 3D). Western blot analysis confirmed increased protein expression levels of these hub genes following SCI, with peak expression observed at days 3–7 post‐injury (Figure 3E). Besides, the mRNA expression levels of these candidate genes were experimentally verified through RT‐qPCR (Figure 3F).

Using the ssGSEA algorithm, we calculated necroptosis phenotype scores based on the expression profiles of these hub genes. The SCI group exhibited significantly higher necroptosis phenotype scores compared to those of controls (Figure 3G, H). To further characterize subgroups, we stratified SCI samples into high‐ and low‐risk categories using the median ssGSEA score as the cutoff threshold. GSEA of these subgroups demonstrated distinct pathway activation patterns (Figure 3I). The high‐riskgroup showed significant enrichment of inflammation‐related pathways, including immune response and cellular response to stimuli, while the low‐risk group exhibited enrichment in pathways associated with protein modification and cell cycle regulation.

### 3.4. Immune Cell Infiltration and Immune Microenvironment in SCI

**Figure 4:**
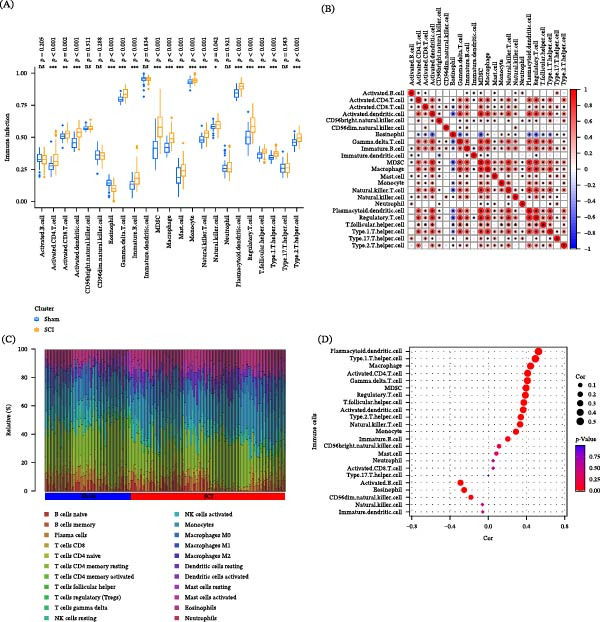
Landscape alterations of immune infiltration in SCI and sham group. (A) Boxplot of immune infiltration levels for various immune cell types across SCI and sham groups, grouped by cluster. (B) Correlation matrix showing the relationships between various immune cell types. Stars indicate statistically significant correlations, with red circles indicating positive correlations and blue circles indicating negative correlations. (C) Stacked bar plot showing the relative proportion of different immune cell types in SCI and sham groups, visualized across multiple clusters. (D) Dot plot depicting the correlation of immune cells with ssGSEA score of key genes. Larger red dots represent stronger positive correlations, while blue dots represent negative correlations, with the size and color gradient indicating the correlation strength and *p*‐value. Data were analyzed with two‐tailed unpaired Student’s *t*‐test (A). Statistical significance is indicated as  ^∗^ *p* < 0.05,  ^∗∗^ *p* < 0.01, and  ^∗∗∗^ *p* < 0.001.

Given the pivotal role of immune cells in SCI pathogenesis, we systematically evaluated immune cell infiltration patterns using a comprehensive panel of 28 immune cell types. Comparative analysis revealed significant differences in 22 immune cell populations between SCI and sham groups, with all showing elevated infiltration levels in the SCI group (Figure 4A). Correlation analysis demonstrated strong interrelationships among these immune cell types, indicating a highly coordinated immune response following SCI (Figure 4B). Further investigation established significant positive correlations between necroptosis activity and immune cell infiltration levels. Notably, plasmacytoid dendritic cells (pDCs), T helper 1 (Th1) cells, and macrophages showed the strongest associations with necroptosis activity, suggesting their potential involvement in necroptosis‐mediated inflammatory responses post‐SCI (Figure 4C, D).

### 3.5. *Tlr4* Upregulates in Myeloid Cells

**Figure 5:**
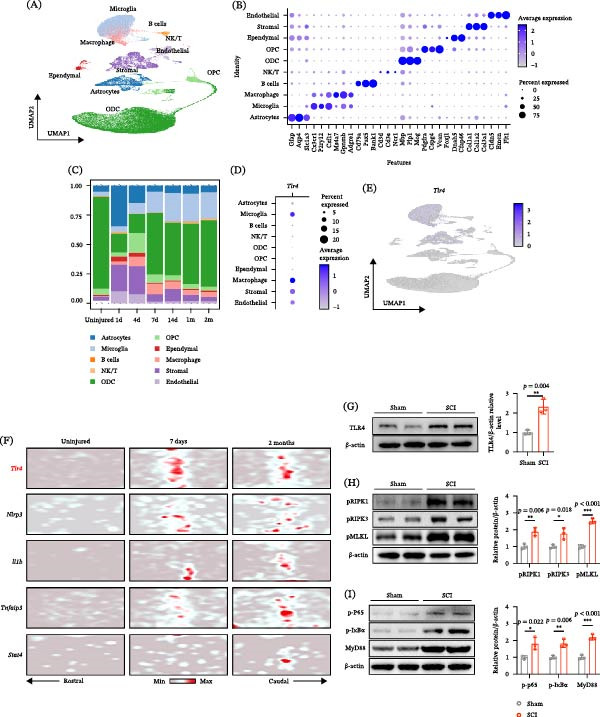
*Tlr4* is upregulated in myeloid cells (microglia and macrophages) after SCI. (A) UMAP plot of single‐cell RNA sequencing (scRNA‐seq) showing the clustering of various cell types in GSE234774. (B) Dot plot representation of cell clusters and their corresponding marker genes. (C) Stacked bar plot showing the proportion of different cell types across different time points, indicating the immune composition of the SCI and sham groups. (D) Dot plot showing the expression of *Tlr4* across different cell types. (E) UMAP plots showing the expression of *Tlr4* across single cells. (F) Visualization of selected gene expression in the ST analysis of sham, 7 days, and 2 months mice. (G) Western blot analysis of Tlr4 protein expression in myeloid cells isolated from spinal cords of sham and SCI mice, with quantification normalized to β‐actin (*n* = 3 per group). (H) Western blot analysis of pRIPK1, pRIPK3, and pMLKL protein expression in myeloid cells isolated from spinal cords of sham and SCI mice, with quantification normalized to β‐actin (*n* = 3 per group). (I) Western blot analysis of p‐p65, p‐IκBα, and MyD88 protein expression in myeloid cells isolated from spinal cords of sham and SCI mice, with quantification normalized to β‐actin (*n* = 3 per group). Data were analyzed with two‐tailed unpaired Student’s *t*‐test (G–I). Statistical significance is indicated as  ^∗^ *p* < 0.05,  ^∗∗^ *p* < 0.01, and  ^∗∗∗^ *p* < 0.001.

We analyzed a subset of the GSE234774 dataset, implementing stringent quality control measures and cell type identification procedures (Figure 5A–C). This analysis identified 10 distinct clusters, and *Tlr4* was predominantly and highly expressed in microglia and macrophages (Figure 5D, E). ST analysis revealed that *Tlr4* exhibited widespread expression within the lesion site and peri‐lesional regions at 7 days post‐injury; notably, by 2 months post‐injury, its spatial distribution became more restricted, with the expression boundary showing a marked retraction toward the lesion core (Figure 5F). Western blotting confirmed upregulation of Tlr4 protein expression in myeloid cells isolated from the spinal cord tissue after SCI (Figure 5G). Finally, we directly assessed the activation of the necroptosis pathway in myeloid cells by measuring pRIPK1, pRIPK3, and pMLKL (Figure 5H). To further validate the proposed *Tlr4*/MyD88/NF‐κB signaling axis, we examined the expression of key molecules in this pathway. As shown in Figure 5I, SCI significantly upregulated MyD88 expression, increased the phosphorylation of IκBα (p‐IκBα), and enhanced the phosphorylation of p65 NF‐κB (p‐p65) in isolated myeloid cells compared to sham controls.

### 3.6. *Tlr4* Deficiency Reduced Neuronal Death by Inhibiting Necroptosis

**Figure 6:**
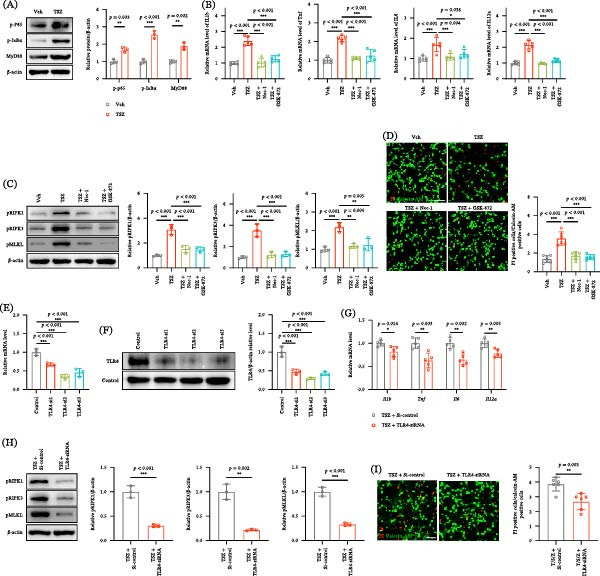
Silencing of *Tlr4* in myeloid cells reduces inflammation and neuronal death associated with necroptosis. (A) Western blot analysis of p‐p65, p‐IκBα, and MyD88 protein expression in myeloid cells with or without TSZ, with quantification normalized to β‐actin (*n* = 3 per group). (B) The mRNA levels of inflammatory cytokines in myeloid cells under vehicle, TSZ treatment, and TSZ treatment followed by rescue with necrostatin 1 (Nec‐1) or GSK‐872 (*n* = 5 per group). (C) Western blot analysis of pRIPK1, pRIPK3, and pMLKL protein expression in myeloid cells under vehicle, TSZ treatment, and TSZ treatment followed by rescue with necrostatin 1 (Nec‐1) or GSK‐872 (*n* = 3 per group). (D) Representative immunofluorescence images of calcein‐AM/PI double staining of neurons after coculture with myeloid cells treated under four conditions: vehicle, TSZ alone, TSZ + Nec 1, and TSZ + GSK‐872, with quantification of calcein‐AM positive (live) and PI positive (dead) neurons (*n* = 5 per group). (E) The efficiency of *Tlr4* siRNAs in myeloid cells detected by RT‐qPCR (*n* = 3 per group). (F) Western blotting for *Tlr4* levels in myeloid cells transfected with Si‐control or Tlr4 siRNAs (*n* = 3 per group). (G) The mRNA levels of inflammatory cytokines in myeloid cells treated with TSZ plus control siRNA (si‐Control) and TSZ plus *Tlr4* siRNA (*n* = 5 per group). (H) Western blot analysis of pRIPK1, pRIPK3, and pMLKL protein expression in myeloid cells treated with TSZ plus control siRNA (si‐Control) and TSZ plus *Tlr4* siRNA (*n* = 3 per group). (I) Representative immunofluorescence images of calcein‐AM/PI double staining of neurons after coculture with myeloid cells treated with TSZ plus control siRNA (si‐Control) or TSZ plus *Tlr4* siRNA, with quantification of calcein‐AM positive (live) and PI positive (dead) neurons (*n* = 6 per group). Data were analyzed with two‐tailed unpaired Student’s *t*‐test (A,G–I) and one‐way analysis of variance followed by post hoc Bonferroni correction (B–F). Statistical significance is indicated as  ^∗^ *p* < 0.05,  ^∗∗^ *p* < 0.01, and  ^∗∗∗^ *p* < 0.001.

We treated myeloid cells with TSZ to simulate necroptosis, as previously described [18]. As expected, treatment with TSZ upregulated MyD88 expression and increased the phosphorylation of IκBα and p65 (Figure 6A). Compared to the vehicle group, TSZ treatment significantly upregulated the mRNA levels of proinflammatory cytokines (*Il1b*, *Tnf*, *Il6*, and *Il12a*), which were subsequently rescued by Nec 1 or GSK‐872 (Figure 6B). Consistently, the protein levels of pRIPK1, pRIPK3, and pMLKL showed a similar pattern (Figure 6C). In addition, TSZ induced neuronal death in the myeloid neuron coculture system, and this effect was also rescued by Nec 1 or GSK‐872 (Figure 6D).

For *Tlr4* siRNA, we observed that siRNA2 effectively suppressed *Tlr4* expression at both the mRNA and protein levels (Figure 6E, F). In the *Tlr4* siRNA group, silencing of *Tlr4* significantly reduced the levels of inflammatory cytokines compared to those of the control group (Figure 6G). Besides, necroptotic markers in myeloid cells demonstrated the inhibitory effect of siRNA on necroptosis (Figure 6H). The coculture of myeloid cells and neurons showed that *Tlr4* silencing eliminated the neuronal destruction ability based on necroptosis (Figure 6I).

### 3.7. Knockout (KO) of *Tlr4* Alleviated SCI by Inhibiting Inflammation In Vivo

**Figure 7:**
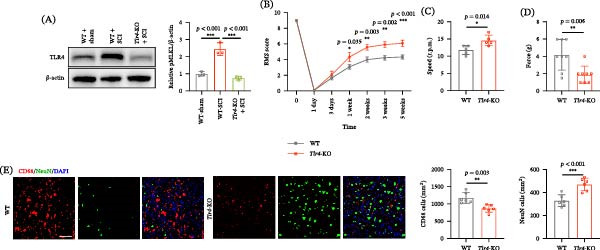
KO of *Tlr4* suppresses inflammation and ameliorates SCI. (A) WB analysis of spinal tissue from WT‐sham, WT‐SCI, and *Tlr4*‐KO + SCI groups, with quantification normalized to β‐actin (*n* = 3 per group). (B) Basso Mouse Scale (BMS) scores for different groups on 0, 1, 3 days, 1, 2, 3, and 5 weeks post‐SCI surgery (*n* = 12 per group). (C) Rotarod test results at day 28 post‐injury for the different groups (*n* = 5 per group). (D) Sensory behaviors by von Frey test at day 28 post‐injury for the different groups (*n* = 8 per group). (E) Representative immunofluorescence images of CD68 and NeuN, along with semiquantitative analysis, from SCI‐WT and SCI‐*Tlr4*‐KO mice 14 days post‐injury (*n* = 6 per group). Data were analyzed with one‐way analysis of variance followed by post‐hoc Bonferroni correction (A), two‐way ANOVA followed by post hoc Bonferroni correction (B) and two‐tailed unpaired Student’s *t*‐test (C–E). Statistical significance is indicated as  ^∗^ *p* < 0.05,  ^∗∗^ *p* < 0.01, and  ^∗∗∗^ *p* < 0.001.

To investigate the regulatory role of *Tlr4* in inflammation following SCI, spinal cord segments from sham, SCI, and SCI–*Tlr4*‐KO mice were collected 7 days after the procedure. WB analysis confirmed the absence of *Tlr4* in the *Tlr4*‐KO group (Figure 7A). Consistently, BMS scores and rotarod test results further demonstrated that *Tlr4*‐KO promoted functional recovery and improved motor coordination after SCI (Figure 7B, C). In addition to motor function, *Tlr4*‐KO also increased tactile sensitivity in injured mice (Figure 7D). Importantly, the *Tlr4*‐KO group exhibited reduced infiltration of inflammatory cells (CD68+) and greater neuronal survival (Figure 7D,E).

## 4. Discussion

Necroptosis is a form of programed cell death that is distinct from apoptosis, characterized by cell membrane rupture, release of cellular contents, and subsequent inflammatory responses [19]. Unlike apoptosis, necroptosis is caspase‐independent and is typically initiated by death receptors, leading to the activation of receptor‐interacting protein kinases (RIPK1 and RIPK3) and the mixed lineage kinase domain‐like protein (MLKL) [10, 12, 20]. Necroptosis has been implicated in the secondary injury phase in SCI, contributing to neuronal loss and exacerbating neurological deficits [21]. Recent studies have demonstrated that necroptosis is not only involved in neuronal cell death but also plays a critical role in the activation and regulation of CNS myeloid cells, including resident microglia and infiltrating macrophages, in the injured spinal cord [22, 23]. Resident microglia and infiltrating macrophages differ in activation kinetics and inflammatory responses after SCI. Microglia are rapidly activated and transition from a proinflammatory to an anti‐inflammatory state, whereas macrophages display a more complex response, exhibiting both proinflammatory and neuroprotective effects, and participate in the acute inflammatory response and tissue repair [24]. In *Tlr4*‐mediated necroptosis, microglia act as direct effector cells, while macrophages may also serve as upstream drivers, releasing inflammatory mediators that cause secondary damage to other neural cells [11]. Collectively, the activation of these myeloid cells leads to the release of proinflammatory cytokines and ROS, which further promote necroptosis and establish a vicious cycle of inflammation and cell death [25]. However, the precise mechanisms by which necroptosis in myeloid cells contributes to SCI pathology remain to be fully elucidated.

We employed WGCNA and differential gene analysis to identify necroptosis‐related DEGs, followed by the LASSO algorithm to screen out five key necroptosis‐associated genes (*Tlr4*, *Nlrp3*, *Il1b*, *Tnfaip3*, and *Stat4*). Phenotypic scores were quantified using the ssGSEA algorithm, enabling the systematic evaluation of necroptosis activity in SCI progression. Through functional enrichment analysis, correlation networks, immune infiltration profiling, and protein interaction mapping, we established a strong association between necroptosis and SCI. Besides, scRNA‐seq further localized the expression of the key gene *Tlr4* predominantly in myeloid cells. Subsequent in vitro experiments demonstrated that myeloid cells with necroptosis could trigger an inflammatory response and destroy neurons. *Tlr4* inhibition significantly reduced neuronal death, highlighting its potential as a therapeutic target by alleviating necrotic‐mediated neuroinflammation.

Through integrative analysis, we identified critical necroptosis‐associated genes, including *Tlr4*, *Il1b*, *Tnfaip3*, *Nlrp3*, and *Stat4*, which are pivotal in regulating inflammatory responses and necroptosis within the CNS. *Tlr4*, highly expressed in myeloid cells, orchestrates innate immune responses by activating NF‐κB and MAPK pathways, thereby linking necroptosis to neuroinflammation in SCI [26, 27]. Tnfaip3 is a critical immunoregulator that suppresses NF‐κB signaling to mitigate neuroinflammation [28]. By inhibiting NF‐κB overactivation, it reduces proinflammatory cytokine release and alleviates secondary tissue damage [29]. The NLRP3 inflammasome, activated by danger signals post‐SCI, synergizes with CASP1 to amplify IL‐1β secretion and necroptotic cell death, contributing to neuronal loss and glial scar formation [30, 31].

Through ST and scRNA‐seq, we validated the spatial expression of necroptosis‐associated genes in the injured spinal cord, with *Tlr4* specifically localized to microglia and macrophages andsignificantly upregulated post‐SCI. *Tlr4* activation was temporally correlated with secondary injury progression. Mechanistically, *Tlr4* engages the NF‐κB pathway in myeloid cells, amplifying proinflammatory cytokine production (e. g., TNF‐α and IL‐1β) and initiating necroptosis via pRIPK1/pRIPK3/pMLKL signaling [32–34]. This cascade exacerbates neuroinflammation and neuronal loss, as evidenced by *Tlr4*‐dependent necroptosis in retinal degeneration and ischemic angiogenesis models [35]. Notably, multiple studies have confirmed that *Tlr4* drives polarization toward a proinflammatory M1 phenotype [36, 37]. Conversely, *Tlr4* inhibition attenuates necroptosis and shifts myeloid cells toward a neuroprotective M2 state, reducing the lesion volume and improving functional recovery [38]. These findings align with recent studies demonstrating that necroptosis is a critical driver of neuroinflammatory cascades in CNS injuries, including SCI and neurodegenerative diseases [39].

It is worth noting that our findings highlight a critical role of *Tlr4*‐mediated necroptosis in the early phases of SCI. This is consistent with previous studies suggesting that inflammatory responses and regulated cell death pathways, including pyroptosis and autophagy, are most active within the acute stage following SCI and tend to subside during the chronic phase [5, 40, 41]. Therefore, *Tlr4* signaling may exert its most pronounced pathological effects during the early post‐injury window. Specifically, we demonstrate that *Tlr4* inhibition reduces pRIPK1, pRIPK3, and pMLKL, thereby linking innate immune signaling to the execution of necroptosis. To our knowledge, this is the first study to establish *Tlr4* as a direct upstream regulator of the pRIPK1/RIPK3/MLKL necroptotic axis in SCI, distinct from its role as a general inflammatory mediator. Our in vivo and in vitro inhibition of *Tlr4* indicates that *Tlr4* represents an attractive therapeutic target due to its upstream role in regulating both necroptosis and neuroinflammation. However, systemic *Tlr4* inhibition may pose significant challenges. Systemic inhibition of *Tlr4* could impair innate immune responses and potentially lead to increased susceptibility to infection or immune dysregulation given its broad role in host defense mechanisms [42, 43]. To overcome these risks, intrathecal administration of *Tlr4* inhibitors has been explored and shows promise for enhancing therapeutic specificity and minimizing off‐target effects [44].

This study has several limitations. First, while *Tlr4* was identified as a key regulator of necroptosis and neuroinflammation in myeloid cells, its interactions with other key genes (e. g., *Nlrp3* and *Il1b*) remain unclear, leaving gaps in understanding the broader immune network in SCI. Second, global *Tlr4*‐KO mice were used in this study, which does not allow us to distinguish the cell type‐specific contributions of *Tlr4*. While our in vitro siRNA data support a role for *Tlr4* in myeloid cells, we cannot rule out the contribution of *Tlr4* deletion in other cell types. Future studies using conditional KO mice are needed to further validate the cell‐specific role of *Tlr4* in SCI. Third, our analysis primarily focused on acute and subacute phases, potentially overlooking the role of *Tlr4*‐mediated necroptosis in chronic glial scar formation and long‐term neurodegeneration. This temporal bias highlights the need for longitudinal studies to fully capture the SCI pathology.

## 5. Conclusions

In conclusion, our findings strongly suggest that *Tlr4*‐mediated necroptosis plays a critical role in the activation and neuroinflammation of myeloid cells following SCI. The key genes and pathways identified, particularly *Tlr4*, exhibit significant diagnostic and therapeutic potential for SCI progression. Furthermore, we demonstrated that this mechanism is predominantly localized to myeloid cells within the injured spinal cord, where *Tlr4* upregulation drives necroptosis and amplifies secondary injury. Targeting the *Tlr4*‐necroptosis axis represents a promising therapeutic target, ultimately advancing the principles of precision medicine in neurotrauma.

## Author Contributions

**Wanzhou Wang**: formal analysis, software, supervision, writing – original draft. **Lu Sun**: software, supervision, validation. **Wei Xie**: project administration, resources. **Fangqing Chen**: investigation, methodology. **Cheng Hong**: conceptualization, data curation, funding acquisition, investigation, methodology, visualization, writing – review and editing.

## Funding

The authors have nothing to report.

## Disclosure

All authors have read the final version and agreed to the submission.

## Ethics Statement

All animal procedures were approved by the animal ethics committee of the Affiliated Geriatric Hospital of Nanjing Medical University (2025‐127‐2) on February 26, 2025. This study complied with the ARRIVE guidelines.

## Conflicts of Interest

The authors declare no conflicts of interest.

## Supporting Information

Additional supporting information can be found online in the Supporting Information section.

## Supporting information


**Supporting Information 1** Table S1: Primers used in qPCR analysis in this study.

**Supporting Information 2** Table S2: The siRNA sequences in this study.

## Data Availability

All the data supporting the findings of this study are publicly available. The datasets GSE5296, GSE47681, GSE42828, and GSE234774 were downloaded from the Gene Expression Omnibus (GEO) database (https://www.ncbi.nlm.nih.gov/geo/). No new data were generated in this study.
