# Supplementary material for: Multiomics Profiling Identifies Tlr4 as a Therapeutic Target of Necroptosis in Spinal Cord Injury
Source: Mediators Inflamm. 2026 Jul 24;2026:7306884. doi: 10.1155/mi/7306884 (PMC13401065; doi:10.1155/mi/7306884)
Supplement: Supplementary file 1 — Supporting Information 1 Table S1: Primers used in qPCR analysis in this study. [file MI-2026-7306884-s001.docx]

**Table S1.** Primers used in qPCR analysis in this study.

| Primer | Sequence (5'-3) |
| --- | --- |
| *Tlr4-F* | ATCCCTGCATAGAGGTAGTTCC |
| *Tlr4-R* | TCAAGGGGTTGAAGCTCAGA |
| *Nlrp3-F* | AGGCTGCTATCTGGAGGAACT |
| *Nlrp3-R* | GCAACGGACACTCGTCATCT |
| *Il1b-F* | TGCCACCTTTTGACAGTGATG |
| *Il1b-R* | AAGGTCCACGGGAAAGACAC |
| *Tnfaip3-F* | CCTGCCAGCAGGTATATGGG |
| *Tnfaip3-R* | GAGGCAGTTTCCATCACCATTG |
| *Stat4-F* | CCATTCGTTCAAGCGTGTCC |
| *Stat4-R* | GAAACACGCCCCAACTGTTC |
| *Tnf-F* | AGGCACTCCCCCAAAAGATG |
| *Tnf-R* | CCACTTGGTGGTTTGTGAGTG |
| *Il6-F* | AGTCCGGAGAGGAGACTTCA |
| *Il6-R* | GTGACTCCAGCTTATCTCTTGGT |
| *Il12a-F* | TCTCACCGTGCACATCCAAG |
| *Il12a-R* | CAGGCAACTCTCGTTCTTGTG |
