# Supplementary material for: Multiomics Profiling Identifies Tlr4 as a Therapeutic Target of Necroptosis in Spinal Cord Injury
Source: Mediators Inflamm. 2026 Jul 24;2026:7306884. doi: 10.1155/mi/7306884 (PMC13401065; doi:10.1155/mi/7306884)
Supplement: Supplementary file 2 — Supporting Information 2 Table S2: The siRNA sequences in this study. [file MI-2026-7306884-s002.docx]

**Table S2.** The siRNA sequences in this study.

| **Gene** | **Sense sequence** | **Antisense-sequence** |
| --- | --- | --- |
| control | UUCUCCGAACGUGUCACGU | ACGUGACACGUUCGGAGAA |
| mTLR4 si-1 | GGUUGCUGUUCUUAUUCUGAU | AUCAGAAUAAGAACAGCAACC |
| mTLR4 si-2 | CCUGUAAGUUACCUGCAUAUU | AAUAUGCAGGUAACUUACAGG |
| mTLR4 si-3 | CCGUUGGUGUAUCUUUGAAUA | UAUUCAAAGAUACACCAACGG |
